# Draft Genome Sequence of* Terrimicrobium sacchariphilum* NM-5^T^, a Facultative Anaerobic Soil Bacterium of the Class* Spartobacteria*

**DOI:** 10.1128/genomeA.00666-17

**Published:** 2017-07-27

**Authors:** Yan-Ling Qiu, Dieter M. Tourlousse, Norihisa Matsuura, Akiko Ohashi, Yuji Sekiguchi

**Affiliations:** a College of Environmental Science and Engineering, Qingdao University, Qingdao, Shandong Province, People's Republic of China; b Biomedical Research Institute, National Institute of Advanced Industrial Science and Technology (AIST), Tsukuba, Ibaraki, Japan; c Faculty of Environmental Design, Kanazawa University, Kanazawa, Ishikawa, Japan

## Abstract

We report here a high-quality draft genome sequence of *Terrimicrobium sacchariphilum* strain NM-5^T^, a facultative anaerobic, mesophilic, fermentative bacterium belonging to the class *Spartobacteria* of the phylum *Verrucomicrobia*. The genome comprises 4,751,807 bp in three contigs and has a G+C content of 60.19%. Annotation predicted 4,175 protein-coding sequences and 54 RNAs.

## GENOME ANNOUNCEMENT

Members of the class *Spartobacteria* (subdivision 2) represent a dominant verrucomicrobial lineage in soil, and they are also ubiquitous in freshwater and marine environments. Their metabolic capacities and ecological roles remain poorly understood, however, due to the paucity of cultured representatives and sequenced genomes. Currently, only two isolates from the class *Spartobacteria* have been described, namely, *Terrimicrobium sacchariphilum* NM-5^T^ (JCM 17479^T^, CGMCC 1.5168^T^) (1) and *Chthoniobacter flavus* Ellin428 (2). Both species share 89.6% 16S rRNA gene sequence identity and display various phenotypic characteristics. *T. sacchariphilum* NM-5^T^ originated from an anoxic rice paddy field and was characterized as a mesophilic anaerobic bacterium utilizing simple carbohydrates for fermentative growth (1). *C. flavus* Ellin428 is an aerobic heterotroph capable of growing on a wider range of carbohydrates derived from plant biomass (2); its genome was sequenced previously (3). Genomes of uncultivated *Spartobacteria* isolates from marine environments (“Spartobacteria baltica”) and soil (“*Candidatus* Udaeobacter copiosus”) were reported more recently (4, 5).

Genomic DNA of *T. sacchariphilum* NM-5^T^ was obtained by phenol-chloroform extraction, and sequencing libraries were constructed with the TruSeq Nano DNA LT library prep kit (450- to 1,600-bp inserts) and the Nextera mate-pair library preparation kit (1- to 16-kb inserts). Sequencing was performed on the Illumina MiSeq platform using v2 chemistry (500 cycles), at coverages of 292× and 133× for the paired-end and mate-pair libraries, respectively. Raw reads were quality filtered using Trimmomatic version 0.32 (6), and the surviving paired-end reads were merged with FLASH version 1.2.11 (7). For the mate-pair library, reads were further processed with NextClip version 1.3.1 (8), and reads assigned to categories A, B, and C were retained. Assembly was performed using SPAdes version 3.6.0 (9), followed by additional scaffolding and manual refinement of the assembly as described previously (10). The Integrated Microbial Genomes (IMG) system (11) was used for genome annotation.

The final draft assembly of *T. sacchariphilum* NM-5^T^ consists of three contigs, with a read coverage of 425×. The total assembly size of *T. sacchariphilum* NM-5^T^ is 4,751,807 bp, which is similar to the mean genome size (4.74 Mb) of soil bacteria (5), and the genome G+C content is 60.19%. Annotation predicted 4,175 protein-coding sequences, 54 RNAs, and 4 rRNAs. The majority of the protein-coding genes (72.55%) were assigned with a putative function and a single complete set of rRNA genes was identified. Of note is that while the gene content of *T. sacchariphilum* NM-5^T^ suggests an aerobic heterotrophic metabolism, the strain was characterized as a strictly anaerobic carbohydrate-fermenting bacterium (1). To clarify this discrepancy, we repeated cultivation experiments to establish that strain NM-5^T^ can grow under aerobic conditions (20% oxygen in the head space) with a range of carbohydrates, including arabinose, fructose, galactose, glucose, ribose, mannose, lactose, maltose, and sucrose, but not with organic acids, alcohols, amino acids, or aromatic compounds, which is consistent with its genomically encoded metabolic potential. We expect that the genome sequence reported here will contribute to improving our understanding of the metabolic capabilities and ecological roles of *Spartobacteria* species in the environment.

### Accession number(s).

The draft genome sequence of *T. sacchariphilum* NM-5^T^ has been deposited at DDBJ/EMBL/GenBank under accession number BDCO00000000 (BioProject PRJDB4647). The version described in this paper is the first version, BDCO01000000.
